# *Hordeum vulgare* L. microgreen mitigates reproductive dysfunction and oxidative stress in streptozotocin‐induced diabetes and aflatoxicosis in male rats

**DOI:** 10.1002/fsn3.2936

**Published:** 2022-07-07

**Authors:** Marwa S. Khattab, Tahany A. A. Aly, Sara M. Mohamed, Abdel Moneim M. Naguib, Ammar AL‐Farga, Emam A. Abdel‐Rahim

**Affiliations:** ^1^ Pathology Department, Faculty of Veterinary Medicine Cairo University Giza Egypt; ^2^ Regional Center for Food and Feed, Agriculture Research Center, Ministry of Agriculture Giza Egypt; ^3^ Biochemistry Department, Faculty of Agriculture Cairo University Giza Egypt; ^4^ Department of Biochemistry, College of Sciences University of Jeddah Jeddah Saudi Arabia

**Keywords:** aflatoxin, barley, chromosomal aberration histopathology, diabetes, sperm analysis

## Abstract

Diabetes mellitus type 2 (DM) is a common chronic disease worldwide, which may be due to increased environmental pollution. Aflatoxin B1 is a likely inevitable contaminant in food and dairy products. Both DM and aflatoxicosis exert a deleterious effect on reproduction urging the exploration of various functional food for protection. This study investigated the effect of barley microgreen (BM) on reproductive disorders caused by DM with or without aflatoxicosis in male rats. Rats were divided into eight groups; G1 control, G2 barley, G3 aflatoxin, G4 aflatoxin‐barley, G5 streptozotocin (STZ), G6 STZ‐barley, G7 STZ‐aflatoxin, and G8 STZ‐aflatoxin‐barley. BM chemical composition revealed elevated calcium, iron, phosphorus, and vitamin A compared with barely seeds. Complete blood picture, lipid profile, serum oxidative stress parameters, relative testicular weight, sperm analysis, chromosomal aberration, and testis histopathology were performed. The lipid profile was altered significantly in G7. Oxidative stress was increased in G3, G5, and G7, whereas it was decreased in BM‐treated groups. Sperm counts were reduced significantly in aflatoxin and/or STZ groups but increased significantly in BM‐treated groups. Sperm morphological abnormalities and chromosomal aberrations were decreased significantly in BM‐treated groups compared with untreated groups. Testicular histopathology revealed moderate diffuse degeneration of seminiferous tubules in aflatoxin and/or STZ groups, which were alleviated in BM‐treated groups. In conclusion, aflatoxin and STZ together caused severe reproductive disorder and oxidative stress more than aflatoxin or STZ alone. BM diet reduced significantly oxidative stress and reproductive disorder associated with DM and aflatoxicosis in rats.

## Graphical abstract

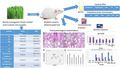

## 1 INTRODUCTION

Environmental toxins were recently associated with increased chronic diseases like diabetes mellitus (DM) (Olokoba, Obateru, & Olokoba, 2012). The progressive escalation in the incidence of diabetes mellitus type 2 (DM) urges further investigation to elucidate the role of environmental toxins in the initiation and deterioration of DM type 2. Exposure to aflatoxin was linked with increased incidence and aggravation of DM. Aflatoxins are a natural toxic contaminant of food crops that are produced by certain kinds of fungi (molds) and threaten human and livestock health (Who, 2018).

Diabetes causes hyperglycemia due to the hindrance of insulin function and/or production. Hyperglycemia disrupts homeostasis in the body with a subsequent oxidative stress complication (Maritim & Saders, 2003). The increase in reactive oxygen species levels and free radicals unsettles the oxidation–reduction balance and thus cellular activities, especially sperm production (Cansian et al., 2015; Dowling & Simmons, 2009; Takao, Imatomi, & Gualtieri, 2015). Unmanaged diabetes with persistent hyperglycemia elicits severe complications like nephropathy, neuropathy, retinopathy, cardiovascular diseases, and male impotency. Furthermore, diabetes alters the gonadal hormone production such as luteinizing, follicle‐stimulating, and testosterone hormones (Ballester et al., 2004). DM alters fat, carbohydrates, and protein metabolism. It causes a wide array of reproductive disorders such as altered spermatogenesis, reduced testosterone and morphological changes in testis as thinning, and premature desquamation of early spermatids and pachytene spermatocytes from the germinal epithelium. These disorders were owed to endocrine disturbances, neuropathy, and increased oxidative stress (Jain & Jangir, 2014; La Vignera, Condorelli, Vicari, D'Agata, & Calogero, 2012). Testicular damage in diabetes occurs mainly due to the release of excessive reactive oxygen species and glycated end products (Yigitturk et al., 2017). Therefore, the use of antioxidants and free radical scavenger supplementations can have a positive effect on spermatogenesis (Shokoohi et al., 2019; Shokoohi et al., 2020).

Reproductive disorders were reported with Aflatoxin B1 in male rats as well (Supriya, Girish, & Reddy, 2014). AFB1 reduces serum testosterone level, lessens sperm quality, and induces germ cell apoptosis (Huang, Cao, Zhang, Ji, & Li, 2019) in addition to decreasing sperm concentration in the epididymis and sperm motility and increasing sperm abnormalities in mice (Agnes & Akbarsha, 2003) and rats (Supriya et al., 2014). Degeneration of seminiferous tubules, sloughing of spermatogenic cells, and complete absence of spermatozoa are the most prominent histopathological lesions of AFB1 toxicity (Abu El‐Saad & Mahmoud, 2009). The AFB1 is metabolized by CYP450 producing DNA adducts and AFB1‐8, 9‐epoxide (Smela, Currier, Bailey, & Essigmann, 2001), which triggers oxidative stress as lipid peroxidation and decreases antioxidant enzymes in affected tissues such as the liver (El‐Bahr, 2015) and testis (Abu El‐Saad & Mahmoud, 2009). CAT and SOD activities and GSH content in rat liver were reduced due to AFB1 injection, which was attributed to the downregulation of gene expression of antioxidant enzymes (El‐Bahr, 2015; El‐Bahr et al., 2015). Activities of antioxidant enzymes were also reduced significantly in testicular tissue (El‐Bahr et al., 2015).

Functional food consumption and a healthy lifestyle provide a promising approach to the prevention and treatment of diabetes. Natural contents of functional food like flavonoids, polyphenols, alkaloids, sterols, pigments, and unsaturated fatty acids possess antioxidant, anti‐inflammatory, and anticholesterol properties and may increase insulin sensitivity (Alkhatib et al., 2017; Supriya et al., 2014). Recently, the consumption of microgreens attracted attention as they have a high nutritional value because they are rich in vitamins (e.g., vitamin C), minerals (e.g., copper and zinc), and phytochemicals, such as phenolic compounds and carotenoids. Microgreens were proved to have antibacterial, anti‐inflammatory, antihyperglycemia, and anticancer properties, which nominate it to be a new functional food (Zhang, Xiao, Ager, Kong, & Tan, 2021). Barley (*Hordeum vulgare* L.) extracts (Minaiyan, Ghannadi, Movahedian, & Hakim‐ Elahi, 2014), leaf (Son, Lee, Park, & Lee, 2016), sprout (Mohamed et al., 2019), seeds (Azam, Itrat, & Ahmed, 2019), grain flour (Abdel‐Gabbar, 2008) and microgreen were recorded to have an antidiabetic potential.

Young barley grass is very rich in minerals like magnesium, sodium, iron, phosphorous, and copper, and vitamins as riboflavin, thiamine, tocopherols, tocotrienols, biotin, pantothenic acid, and folic acid making it superior to some known vegetables (spinach, tomato, lettuce; El‐Dreny & El‐Hadidy, 2018). It also contains high glutamic acid, aspartic acid, ascorbic acid, glucose, and fructose, Gamma‐aminobutyric acid (GABA), phenolic acids, β‐glucan, flavonoids, and phytosterols making it the best functional food for the prevention of chronic diseases like diabetes, cancer, obesity, cardiovascular disease (Zeng et al., 2018) and testicular degeneration as observed in acrylamide toxicity (Abd El‐Aziem, Mahrous, Abdel‐Wahab, Mahmoud, & Hassan, 2004). The mechanism of anti‐inflammatory and cardiovascular disease prevention of barley was accredited to the inhibition of both cyclooxygenase and lipoxygenase pathways of arachidonic acid metabolism and increased activities of superoxide dismutase (SOD) and glutathione peroxidase (GPx) (Gul et al., 2014).

Therefore, this study investigates the effect of barley microgreen as a functional food in the prevention of reproductive disorders associated with diabetes and aflatoxicosis in male rats.

## 2 MATERIALS AND METHODS

### 2.1 Microgreens of barley

Microgreens of barley (BM) (*Hordeum vulgare*) in the fully expended green cotyledons stage were grown in an open field and harvested after 14 days of seed soaking. At this time, the first true leaves start to emerge and contain the highest content of high‐value nutrients with health benefits such as amino acids, enzymes, vitamins, minerals, phenolics, antioxidants, and pigments (Benincasa et al., 2015). Microgreen was washed, hulled, air‐dried for 3 days, and crushed into powder (Abdallah, 2008; Dzowela, Hove, & Mafongoya, 1995).

### 2.2 Chemical composition of barley microgreen

Minerals and vitamins were determined in BM and barley seeds. Calcium (Ca), magnesium (Mg), iron (Fe), phosphorus (P), potassium (K), and zinc (Zn) were analyzed according to AOAC, 2000. Vitamin C (ascorbic acid), vitamin E (tocopherol), and vitamin A (β‐carotene) were determined according to previous methods (Bajaj & Kaur, 1981; Leth & Jacobsen, 1993; Leth & Sondergaro, 1983).

### 2.3 Aflatoxin preparation

*Aspergillus flavus strain* (NRRL 3357) was obtained from the Laboratory of Mycotoxin, National Research Center (Dokki, Giza, Egypt). Under the complete aseptic condition, the lyophilized strain of *Aspergillus flavus* was reconstituted on slants of Czapek's agar media with pH 6.5–6.8 and incubated at 25–29°C for 9 days (Davis, Diener, & Eldridge, 1966). It was then transferred to an autoclaved 2 L (15 min at 121°C) clean sterilized flask containing 250 ml prepared liquid yeast medium (YES) and incubated for 9 days at 25–29°C. To remove the mycelial mats, the medium was filtrated by filter paper. The filtrate was kept at 4°C for later use in tightly wrapped bottles by aluminum foil.

### 2.4 Animals

The animals were purchased from the National Research Center (El Dokki, El Giza, Egypt) and housed in plastic cages (3 rats per cage) at 25 ± 2°C and with humidity of 50–60%. They were kept 2 weeks before the beginning of the experiment for acclimatization. Animals were fed a free‐access pelleted diet and had free access to water.

### 2.5 Diets and their preparation

Four different diets were formulated into pellets; a control diet according to the AIN‐76, a BM diet with 10% BM powder replacing corn starch, an high‐fat diet (HFD) with 20% palm oil instead of corn starch, with HF and BM diet with 20% palm oil and 10% BM on the account of corn starch.

### 2.6 Induction of type 2 diabetes mellitus

Rats were fed HFD ad libitum for 2 weeks and then injected intraperitoneal with a single low dose of streptozotocin (STZ) (30 mg/kg) to induce Type 2 DM (Zhang, Lv, Li, Xu, & Chen, 2008). The fasting blood glucose levels of all rats were measured after 7 days of STZ injection. Rats having blood glucose levels ≥200 mg/dl were considered diabetic and were selected for further experiment and maintained on HFD until the end of the experimental period.

### 2.7 Experimental design

Forty‐eight male albino rats were randomly allocated into 8 groups (6 rats each) in which G1: control rats, G2: rats fed (BM) diet, G3: rats received aflatoxin (30 μg/kg AFB1) 3 days/week orally, G4: received aflatoxin and fed BM, G5: diabetic rats fed a high‐fat diet (HFD), G6: diabetic rats fed HFD with BM, G7: diabetic rats fed HFD and administered aflatoxin, and G8: rats are diabetic rats fed HFD with BM and received aflatoxin orally. The experiment ended after 6 weeks in which serum, blood, and tissue samples were collected.

Group 1 = Negative control group.
Group 2 = Egyptian barley microgreen (EBM) group.
Group 3 = Aflatoxin (30 μg/kg) group.
Group 4 = Aflatoxin + EBM.
Group 5 = Positive diabetic rats (STZ 30 mg/kg) and fed a high‐fat diet (HFD).
Group 6 = HFD and Diabetic + EBM.
Group 7 = Aflatoxin, HFD, and STZ.
Group 8 = Aflatoxin, HFD, and STZ + EBM.

### 2.8 Complete blood picture

Whole blood was collected from the retro‐orbital plexus of veins using micro heparinized tubes. A complete blood picture was carried out using an Automated Hematology Analyzer XT‐2000 il XT‐1800 i. (Sysmex Co., Kobe, Japan).

### 2.9 Determination of lipid profile

Serum triglycerides and total cholesterol were determined colorimetrically according to previous methods (Fossati & Prencipe, 1982; Richmond, 1973). HDL‐cholesterol and LDL‐cholesterol were estimated by the enzymatic colorimetric method (Burstein, Scholnick, & Morfin, 1970; Wieland & Seidel, 1983), and vLDL‐cholesterol was calculated as equation (vLDL = TG/5; Friedewald, Levy, & Fredrickson, 1972).

### 2.10 Determination of serum oxidative stress biomarkers

Reduced glutathione (GSH) determination was based on the reduction of 5.5 dithiobis (2‐nitrobenzoic acid) (DTNB) with glutathione (GSH) to produce a yellow compound, which was calorimetrically determined (Beutler, Duron, & Kelly, 1963). Superoxide dismutase (SOD) activity enzymes are metalloenzymes that catalyze the dismutase of the superoxide anion to molecular oxygen and hydrogen peroxide defense mechanism. SOD was determined according to the previous method (Nishikimi, Rao, & Yagi, 1972). The activity of serum catalase (CAT) (Aebi, 1984), gamma‐glutamyl transferase (ɤGT) (Szasz, 1969), glutathione transferase (GST) (Hebig, Pabst, & Jakoby, 1974), and malonaldehyde (MDA) concentration were measured. MDA concentration was determined based on the reaction with thiobarbituric acid (TBA) reactive substances (TBARS) absorbance of the resultant pink product was determined at 534 nm was measured according to a previous study (Ohkawa, Ohish, & Yagi, 1979).

### 2.11 Body weight and relative testicular weight

The animals were weighed at the end of the experiment to record their body weight. The testis of each rat was excised, blotted, and weighed, and then, the organ/initial body weight ratio was calculated.

### 2.12 Sperm analysis

Sperm analysis was performed in euthanized animals, and the rats were sacrificed after 35 days of the first treatment. For sperm‐shaped analysis, the epididymis was excised and minced in about 10 ml of physiological saline, dispersed, and filtered to exclude large tissue fragments. Smears were prepared after staining the sperms with Eosin Y (aqueous), according to the method of (Wyrobek & Bruce, 1978; Wyrobek, Watchmaker, & Gordon, 1984). At least 4000 sperms per group were assessed for morphological abnormalities. Epididymal sperm count was also determined by a hemocytometer.

### 2.13 Chromosomal analysis

The rats were sacrificed after 15 days of the first induction then rats were studies of chromosomal aberration analysis. Femur bones were collected from euthanized animals and the bone marrow was pooled with 0.9% saline in a tube. Bone marrow metaphases were prepared according to (Yosida, Truchiya, & Moriwaki, 1977) and stained with phosphate‐buffered. Chromosomal aberrations such as chromosomal for chromatid gap, break, deletion, and centromeric attenuation were recorded in at roast 50 well metaphase spread for each animal. The mitotic activity of bone marrow cells was determined for each treated and control animal. It is expressed by the mitotic CMI: No of dividing cells into 1000 cells.

### 2.14 Histopathology

Tissue specimens from the testis of rats at the end of the experiment were fixed in 10% neutral buffered formalin. Specimens were then processed, embedded in paraffin, sectioned (3–4 μm), and stained by hematoxylin and eosin stain (Suvarna, Layton, & Bancroft, 2012). Tissue slides were examined by light microscopy and photographed using a digital camera (Olympus XC30, Tokyo, Japan). The epithelium thickness lining seminiferous tubules were determined using TS view software from the basement membrane to the lumen in 10 tubules/testis at an angle of 90 degrees to calculate the mean of epithelial thickness/rat.

The histopathological changes of spermatogenesis in 10 seminiferous tubules were graded using Johnsens' score on a scale from 1 to 10 (Abdelatty et al., 2020; Johnsen, 1970) Seminiferous tubules showing no seminiferous epithelium are scored 1, presence of Sertoli cells only and no germinal cells are scored 2, presence of spermatogonia only was scored 3, few spermatocytes with no spermatozoa or spermatids was scored 4, many spermatocytes with no spermatozoa or spermatids is scored 5, few early spermatids with no spermatozoa and no late spermatids are scored 6, many early spermatids with no spermatozoa and no late spermatids are scored 7, few late spermatids and less than five spermatozoa per tubule is scored 8, many late spermatids, disorganized epithelium indicating slightly impaired spermatogenesis is scored 9, full spermatogenesis and perfect tubules is scored 10.

### 2.15 Statistical analysis

The size of the sample was calculated according to a previous article (Charan & Biswas, 2013). The Power of a study that is the probability of finding an effect was kept at 80%. The data were tested for homogeneity of variances and analyzed by one‐way ANOVA of statistical package SPSS, version 8.0 (SPSS Inc., Chicago, IL, U.S.A.) followed by post hoc tests (Duncan and Tamhne's tests). A significance was considered at *p* < 0.05. The Johnsen score of spermatogenesis was analyzed by using a nonparametric Kruskal–Wallis test to detect significance at *p* ≤ 0.05. Significant parameters were analyzed by the Mann–Whitney test to show the significance between groups.

## 3 RESULTS AND DISCUSSION

Reproductive disorders were reported previously due to aflatoxicosis and diabetes mellitus; however, the mutual effect of both was not documented before (La Vignera et al., 2012; Supriya et al., 2014) Therefore, the present study shows the mutual deleterious effect of aflatoxicosis and diabetes mellitus on the lipid profile, serum oxidative stress parameters, spermatogenesis, chromosomal aberrations, and testicular histopathology in rats, in addition to investigating the possible protective effect of barley microgreen feeding on aflatoxicosis and diabetes mellitus.

### 3.1 Barely microgreen composition

Barley microgreen (BM) after 14 days of germination showed an increase in all of the evaluated elements except zinc (21.23 mg/100 g) and magnesium (259.8 ± 2.20 mg/100 g) relative to those of dry seeds. Potassium, on the other hand, was decreased in BM compared with their seeds. (Table 1).

**TABLE 1:** Microgreen germination effects on element content and vitamin content (mg/kg dry weight) of the studied Egyptian barley

Samples	Barley seeds	EBM
Ca	254.3 ± 1.80	1002 ± 2.00*
Fe	158.6 ± 2.00	506.1 ± 2.00*
Mg	230.6 ± 1.80	259.8 ± 2.20*
P	1.71 ± 0.20	2500 ± 50.00*
K	649.4 ± 2.20*	42.21 ± 1.80
Zn	17.14 ± 2.00	21.23 ± 2.11*
(β‐carotene) Vitamin A	5.00 ± 0.03	3824.7 ± 16.71*
(Ascorbic acid) Vitamin C	5.12 ± 0.07*	1.41 ± 0.03
(α‐Tocopherol) Vitamin E	1.10 ± 0.05	1.33 ± 0.06*

Abbreviations: BM, barley microgreen; STZ, streptozotocin.

*Note*: All values are represented as mean ± S.D.

Means bearing asterisk are significantly different (*p* < 0.05).

The antioxidant bioactive compounds in fresh microgreens relative to dry seeds showed an increase in vitamin A value (expressed as its precursor β‐carotene) from 5.0 to 3824.7 mg/kg and vitamin E content (α‐tocopherol form) in BM. On the other hand, BM showed a decrease in vitamin C values relative to their dry seeds. (Table 1).

### 3.2 Complete blood picture

The mean corpuscular volume (MCV), mean corpuscular hemoglobin (MCH), mean corpuscular hemoglobin concentrate (MCHC), and red blood cell distribution width (RDW) were not significantly different between groups. On the other hand, the WBCs were significantly increased in the G3 aflatoxin group compared with the control, whereas it was significantly decreased in G4. Platelet count was significantly decreased in G3 but returned to normal level in G4. (Tables 2 and 3).

**TABLE 2:** Complete blood picture in different experimental groups

Treatment	MCV	MCH	MCHC	RDW
	(fl)	(Pg)	(g/dl)	(fl)
G1 control	59.38 ± 0.61^a^	23.15 ± 0.44^a^	38.88 ± 0.70^a^	14.78 ± 0.95^a^
G2 BM	60.40 ± 0.68^a^	22.90 ± 0.34^a^	37.98 ± 0.13^a^	13.78 ± 0.05^a^
G3 aflatoxin	60.68 ± 0.24^a^	23.30 ± 0.08^a^	38.48 ± 0.13^a^	14.28 ± 0.33^a^
G4 aflatoxin BM	60.28 ± 0.10^a^	22.88 ± 0.25^a^	38.13 ± 0.61^a^	14.47 ± 0.19^a^
G5 STZ	61.78 ± 3.00^a^	23.88 ± 0.74^a^	38.78 ± 1.07^a^	15.23 ± 1.33^a^
G6 STZ‐BM	62.09 ± 2.52^a^	23.13 ± 0.47^a^	37.38 ± 0.79^a^	14.95 ± 0.40^a^
G7 STZ‐aflatoxin	61.71 ± 0.39^a^	23.45 ± 0.19^a^	38.40 ± 0.30^a^	14.85 ± 0.34^a^
G8 STZ‐aflatoxin‐BM	60.48 ± 0.38^a^	23.25 ± 0.42^a^	38.53 ± 0078^a^	14.75 ± 1.61^a^

Abbreviations: BM, barley microgreen; STZ, streptozotocin. All values are represented as mean ± S.D.

*Note*: Means with different letters are significantly different (*p* < 0.05).

**TABLE 3:** Hb, HCT, RBCs, WBCs, and PLT in different experimental groups

Treatment	HB (g/dl)	HCT %	RBCs (10^6^/ul)	WBCs (10^3^/ul)	PLT (10^3^/ul)
G1 control	14.63 ± 0.15^a^	37.58 ± 1.0^a^	6.30 ± 0.10^a^	6.85 ± 1.66^c^	653.38 ± 53.76^a^
G2 BM	14.55 ± 0.51^a^	38.25 ± 1.29^a^	6.34 ± 0.21^a^	6.65 ± 2.15^c^	582.00 ± 60.71^b^
G3 aflatoxin	15.23 ± 0.30^a^	39.50 ± 0.73^a^	6.55 ± 0.13^a^	10.64 ± 1.59^a^	480.50 ± 30.82^c^
G4 aflatoxin‐BM	14.51 ± 0.38^a^	38.38 ± 0.21^a^	6.36 ± 0.09^a^	8.40 ± 1.25^b^	651.50 ± 128.99^a^
G5 STZ	15.15 ± 0.93^a^	39.08 ± 3.06^a^	6.33 ± 0.20^a^	6.90 ± 6.07^c^	528.00 ± 155.23^bc^
G6 STZ‐BM	15.16 ± 0.39^a^	40.93 ± 2.54^a^	6.55 ± 0.13^a^	8.19 ± 0.50^b^	521.50 ± 28.80^bc^
G7 STZ‐aflatoxin	15.86 ± 0.43^a^	41.03 ± 0.81^a^	6.71 ± 0.17^a^	5.97 ± 1.24^d^	634.81 ± 161.82^a^
G8 STZ‐aflatoxin BM	15.68 ± 0.61^a^	40.68 ± 2.36^a^	6.74 ± 0.38^a^	7.95 ± 1.81^b^	590.25 ± 70.23^b^

Abbreviations: BM = barley microgreen. All values are represented as mean ± S.D.

*Note*: Means with different letters are significantly different (*p* < 0.05).

Although the differential count of WBCs was not performed in this study, it showed an increase in WBCs. A previous study reported an elevation of neutrophil count in addition to lymphopenia and monocytopenia with AFB1 exposure (Dönmez, Dönmez, Keskin, & Kısadere, 2012). The effect of AFB1 whether stimulatory or suppressive on the immune system varies according to dose and time (Hinton et al., 2003). Aflatoxicated rats on the other hand in the groups fed BM diet showed less alteration in hematological parameters. On the reverse to prior studies, which recorded changes in hematological parameters in diabetic patients, diabetic rats (STZ group) showed no alteration in the present study (Gkrania‐Klotsas et al., 2010).

### 3.3 Lipid profile

The cholesterol, triglycerides, LDL‐c, and vLDL‐c were significantly elevated in the G7 (STZ/aflatoxin group) compared with all other groups. In G8 (STZ/aflatoxin treated with BM), the cholesterol was decreased compared with G7 but recorded no significant difference. HDL was decreased in G3 (aflatoxin control) compared with G1 and G2. All intoxicated groups treated with BM had a decreased LDL‐c concentration (Table 4).

**TABLE 4:** Lipid profile of rats in different groups

Treatment	Cholesterol (mg/dl)	Triglycerides (mg/dl)	HDL (mg/dl)	LDL (mg/dl)	VLDL (mg/dl)
G1 control	141.53 ± 5.12^e^	216.85 ± 7.71^d^	43.33 ± 3.81^ab^	40.67 ± 2.17^b^	43.37 ± 2.78^d^
G2 BM	140.37 ± 7.85^e^	241.10 ± 4.52^c^	45.33 ± 2.36^a^	32.78 ± 2.02^c^	48.22 ± 3.12^cd^
G3 aflatoxin	148.27 ± 3.13^d^	236.00 ± 7.60^c^	38.00 ± 2.92^b^	48.25 ± 3.11^a^	47.2 ± 3.09^cd^
G4 aflatoxin ‐BM	140.61 ± 5.19^e^	230.60 ± 3.53^c^	46.50 ± 2.29^a^	33.93 ± 2.21^c^	46.12 ± 3.21^d^
G5 STZ	158.24 ± 8.42^c^	246.85 ± 3.64^c^	45.17 ± 3.75^a^	47.87 ± 3.02^a^	49.37 ± 3.13^cd^
G6 STZ‐BM	145.54 ± 7.28^de^	281.60 ± 4.96^b^	42.33 ± 1.44^ab^	32.34 ± 2.10^c^	56.32 ± 3.71^c^
G7 STZ‐aflatoxin	205.99 ± 6.21^a^	479.15 ± 10.87^a^	40.00 ± 1.00^ab^	49.56 ± 3.33^a^	95.83 ± 5.67^a^
G8 STZ‐aflatoxin‐BM	187.85 ± 10.21^b^	454.15 ± 7.15^a^	47.33 ± 2.89^a^	34.91 ± 2.14^c^	90.83 ± 6.02^ab^

Abbreviations: BM, barley microgreen; STZ, streptozotocin. All values are represented as mean ± S.D.

*Note*: Means with different letters are significantly different (*p* < 0.05).

The alteration in lipid profile highlights the interplay of aflatoxicosis and diabetes on lipid metabolism. AFB1 acute exposure elevates cholesterol, triglycerides, and phospholipids due to dysregulation of lipid and lipoprotein metabolizing gene expression (Rotimi et al., 2017). In the present study, aflatoxicosis alone showed insignificant alteration in lipid profile except for LDL‐c, which might be due to the small dose used in the current study. Similarly, diabetes alone did not alter the lipid profile significantly except for the LDL, which is compatible with previous studies (Abdel‐Mobdy, Khattab, Mahmoud, Mohamed, & Abdel‐Rahim, 2021; Adedeji & Orisadiran, 2020). In intoxicated group‐fed BM diet, the lipid profile was almost returned to normal. The hypolipidemic effect of an antioxidant such as barley β‐glucan was also recorded previously (Swelim, Farid, & Mostafa, 2019).

### 3.4 Serum oxidative stress parameters

The activity of serum ɤGT was significantly elevated in G7, G3, and G5, respectively. On the other hand, treated groups receiving BM diet (G4, G6, G8) showed a significant improvement and decrease in serum ɤGT compared with their counterparts but was still significantly elevated compared with the G1 control group. Lipid peroxidation was significantly elevated, and GSH content was significantly decreased in intoxicated all treated groups compared to the control group with the highest increase in G7 (STZ‐aflatoxin group) followed by G3 (aflatoxin). The groups receiving BM diet (G4, G6, and G8) recorded a significant decrease in MDA concentration and a significant increase in GSH content compared with their counterparts. Furthermore, the activity of antioxidant enzymes (SOD, CAT, and GST) was significantly reduced in intoxicated groups compared with control, whereas it was significantly restored in part in the groups receiving BM diet compared with their counterparts (Table 5). Under normal physiological conditions, a delicate balance exists between the rate of H_2_O_2_ synthesis via dismutation of O_2_ by SOD activity and the rate of removal of H_2_O_2_ by CAT. Therefore, any impairment in this pathway will affect the activities of other antioxidative enzymes in the cascade (Kon & Fridorich, 1992). The oxidative stress induced by aflatoxin and STZ might be due to their lipophilicity, whereby they can penetrate easily into the cell membrane and cause membrane lipid peroxidation (Supriya et al., 2014; Yigitturk et al., 2017). The treatment with BM as an antioxidant diet alleviated the in vivo effects of aflatoxin and STZ by scavenging neutralizing reactive oxygen species (ROS).

**TABLE 5:** Oxidative stress biomarkers in serum of rats in different experimental groups

Treatment	Activity	MDA	GSH content	Enzyme activity		
	ɤGT	Nmol/ml	μmol/ml	SOD (U/ml)	CAT (U/ml)	GST (μmol/m)
G1 control	31 ± 2.00^e^	0.121 ± 0.011^e^	0.511 ± 0.033^a^	351.11 ± 21.01^a^	8.11 ± 0.51^a^	4.13 ± 0.27^a^
G2 BM	30 ± 2.01^e^	0.114 ± 0.011^e^	0.523 ± 0.028^a^	361.12 ± 22.22^a^	8.12 ± 0.38^a^	4.20 ± 0.27^a^
G3 aflatoxin	158 ± 8.12^b^	0.601 ± 0.042^b^	0.290 ± 0.018^c^	211.22 ± 15.51^e^	5.24 ± 0.31^e^	2.42 ± 0.18^d^
G4 aflatoxin BM	98 ± 5.92^c^	0.389 ± 0.021^c^	0.363 ± 0.022^b^	268.12 ± 17.17^c^	6.17 ± 0.41^c^	2.86 ± 0.17^c^
G5 STZ	81 ± 5.44^d^	0.351 ± 0.021^cd^	0.332 ± 0.020^c^	222.17 ± 16.17^e^	6.11 ± 0.42^c^	2.88 ± 0.14^c^
G6 STZ‐BM	70 ± 4.94^d^	0.288 ± 0.021^d^	0.411 ± 0.031^b^	299.13 ± 18.26^b^	7.30 ± 0.38^b^	3.33 ± 0.21^b^
G7 STZ‐aflatoxin	251 ± 14.11^a^	1.000 ± 0.061^a^	0.211 ± 0.018^d^	200.12 ± 14.44^e^	4.33 ± 0.29^d^	2.11 ± 0.17^e^
G8 STZ‐aflatoxin‐BM	160 ± 10.11^b^	0.487 ± 0.031^b^	0.330 ± 0.017^c^	240.06 ± 13^d^	5.01 ± 0.32^e^	2.52 ± 0.16^d^

Abbreviations: BM, barley microgreen; STZ, streptozotocin. All values are represented as mean ± S.D.

*Note*: Means with different letters are significantly different (*p* < 0.05).

### 3.5 Spermatogenic damage

**FIGURE 1:**
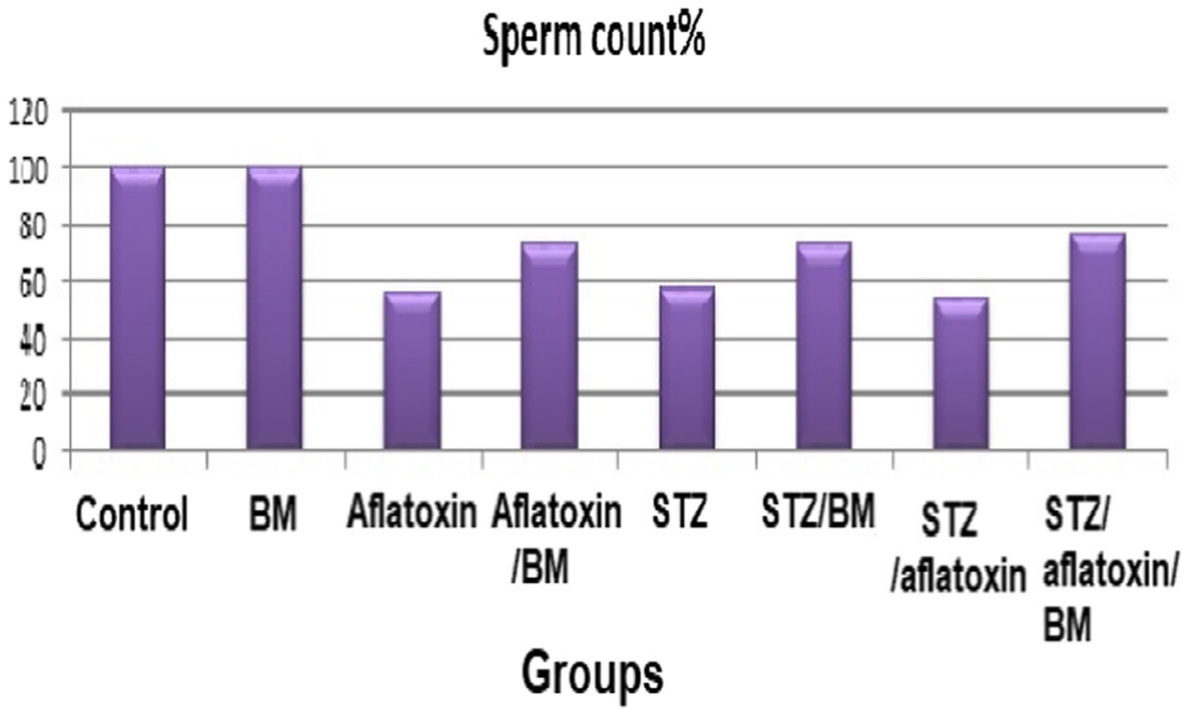
Spermatogenic damage in different groups. BM, Barley microgreen; STZ, Streptozotocin

The testes' weight/body weight ratio was decreased significantly in all intoxicated treated groups (G3, G4, G5, G6, G7, and G8) relative to health control (G1 and G2). This means that the induction with STZ and aflatoxin produced a harmful leanness as emaciation in the testes tissue (G3, G5, and G7). The same trend was observed in the sperm counts for the same three induced groups. The sperm count was reduced significantly in G7 (STZ and aflatoxin), G3 (aflatoxin), and G5 (STZ), respectively, compared with the control. The sperm count was improved and increased significantly in intoxicated groups treated with barley (G4, G6, and G8) compared with intoxicated untreated groups (G7, G3, and G5) (Table 6, Figure 1). These results confirmed each other. The present harmful effect of STZ and aflatoxin on testis was attenuated after the treatment with BM (G4, G6, and G8), but it was slightly lower than those of both healthy groups (G1 and G2).

**TABLE 6:** Spermatogenic damage in aflatoxin intoxicated treated animals

Treatment	Bodyweight (B.W)	Testes weight (T.W)	TW/BW Ratio	Sperm count
	G	g	g/100 g	Value ×10^**6**^
G1 control	118.8 ± 8.9	3.81 ± 0.21	3.21	52.00 ± 2.61a
G2 BM	111.42 ± 8.71	3.87 ± 0.18	3.47	51.76 ± 31.21a
G3 aflatoxin	124.4 ± 10.1	2.00 ± 0.13	1.61	29.66 ± 2.06c
G4 aflatoxin BM	124.83 ± 8.07	2.28 ± 0.11	1.83	37.91 ± 2.11b
G5 STZ	124.8 ± 11.39	2.10 ± 0.13	1.68	30.11 ± 1.98c
G6 STZ‐BM	119.37 ± 10.11	2.40 ± 0.13	2.01	38.11 ± 2.11b
G7 STZ‐aflatoxin	135.73 ± 11.21	1.97 ± 0.11	1.45	28.12 ± 1.88c
G8 STZ‐aflatoxin‐BM	129.21 ± 11.01	2.07 ± 0.13	1.60	40.01 ± 2.99b

Abbreviations: BM, barley microgreen; STZ, streptozotocin. All values are represented as mean ± S.D.

*Note*: Means with different letters are significantly different (*p* < 0.05).

**FIGURE 2:**
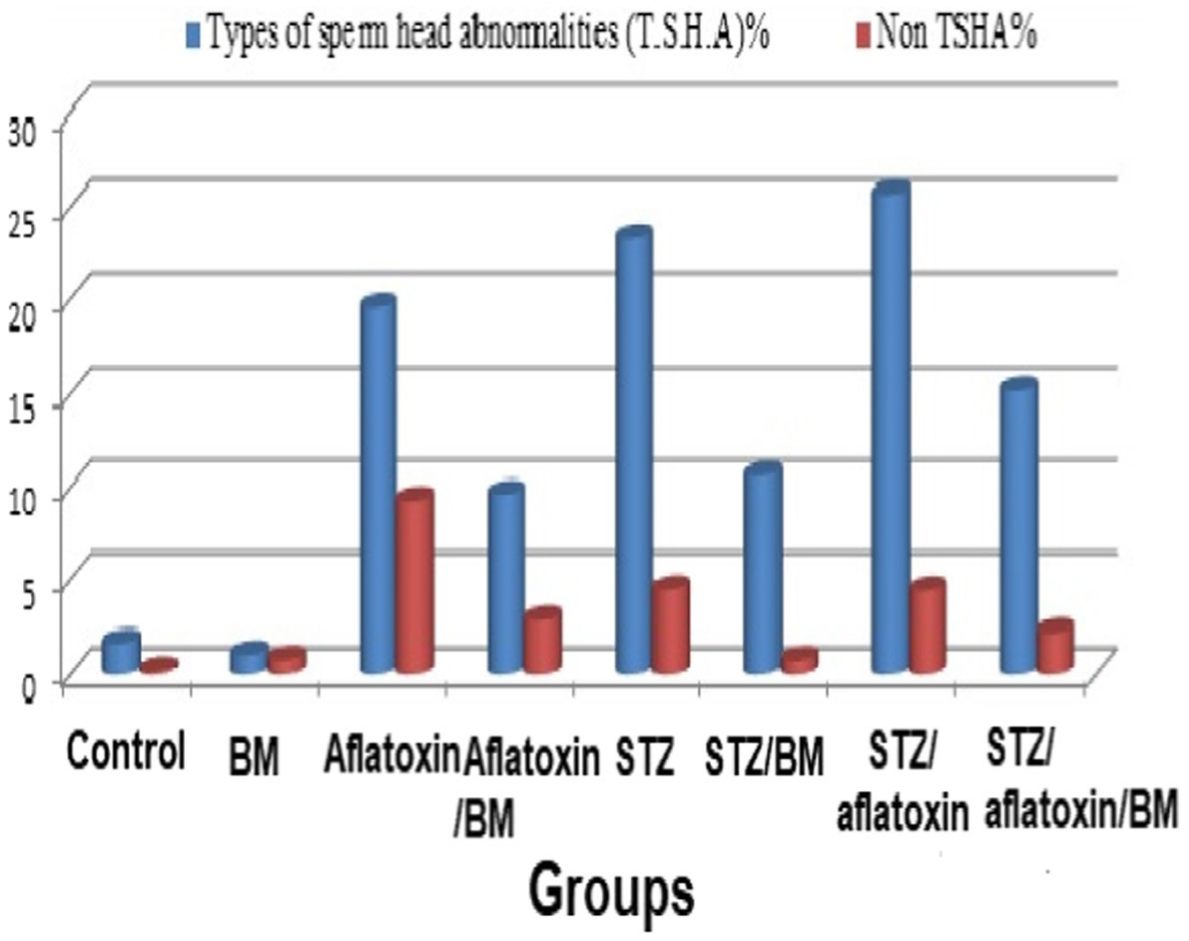
Frequencies of sperm abnormality in the different groups. BM, Barley microgreen; STZ, Streptozotocin

In addition, the results of spermatocytes examination for structural and numerical abnormalities of all studied groups were presented in Table 5, which showed the frequencies of sperm abnormality/4000 sperm examined in the intoxicated and treated rats. The results showed more frequent abnormalities of sperms in the head and tail than those of healthy control and BM‐treated groups. Oral feeding of BM reduced the percentage of abnormal sperms, but their frequencies are still exceedingly significant to those of healthy control rats. There are differences in the total number of abnormal sperms between the three intoxicated groups and the three intoxicated/treated rats with BM. Head abnormalities were increased significantly in G7, G5, and G3 groups compared with the control group, whereas it decreased in intoxicated BM‐treated groups (G4, G6, and G8) but was still higher than the control. (Table 7, Figure 2) Therefore, relative testicular weight, sperm count, and sperm abnormalities count were all affected negatively by aflatoxicosis and diabetes in the current study similar to other research (Jain & Jangir, 2014; Owumi, Adedara, Akomolafe, Farombi, & Oyelere, 2020). These sperm abnormalities indicate a point of mutation in germ cells (Narayna, D'Souza, & Rao, 2002), which could have affected the normal spermatogenesis. This could be due to oxidative stress induced by increased ROS generation, which in turn activates apoptosis and increases DNA damage (Meštrović et al., 2014; Roshangar, Rad, & Afsordeh, 2010; Roushangar & Rad, 2007; Volpe, Villar‐Delfino, Anjos, & Nogueira‐Machado, 2018). The use of plant‐derived antioxidants as polyphenols and flavonoids yielded promising results in lowering the oxidative stress and treatment of male infertility (Shokoohi et al., 2018). Similarly, natural antioxidants alleviated the oxidative stress associated with ovarian damage and improved female fertility (Delkhosh et al., 2019; Shokri, Shokoohi, Abadi, & Kalarestaghi, 2019).

**TABLE 7:** Frequencies of sperm abnormality/4000 sperm examined in the experimental intoxicated and treated animals

Treatment	Abnormal sperm		Types of sperm head abnormalities (T.S.H.A)							Non‐TSHA	
	No	% of 4000	Amorphous	Banana shape	Without hook	Big	Small	Total no	% of 4000	Total no	% at 4000
G1 control	76 ± 4.44^e^	1.90	20 ± 1.21	16 ± 1.01	20 ± 1.11	1±0.01	11 ± 0.71	68 ± 4.11^d^	1.7	8^e^	0.20
G2 BM	75 ± 5.12^e^	1.01	20 ± 1.22	14 ± 1.02	18 ± 1.43	2 ± 0.01	9 ± 0.11	43 ± 3.42^d^	1.08	32^d^	0.80
G3 aflatoxin	1151 ± 54^a^	28.78	401 ± 30.11	250 ± 17.23	99 ± 5.55	131 ± 7.34	101 ± 6.16	781 ± 42.24^a^	19.53	370^b^	9.25
G4 aflatoxin‐ BM	501 ± 31^c^	12.53	125 ± 7.12	70 ± 4.03	64 ± 3.99	28 ± 1.78	97 ± 5.36	384 ± 20.01^c^	9.60	117^c^	2.93
G5 STZ	1111 ± 63^a^	27.78	391 ± 20.22	243 ± 14.26	88 ± 5.21	122 ± 7.37	90 ± 5.12	934 ± 51.11^a^	23.25	177^a^	4.43
G6 STZ‐BM	454 ± 31^d^	11.35	126 ± 6.66	45 ± 2.94	62 ± 4.10	56 ± 3.27	134 ± 8.00	423 ± 27.12^c^	10.58	31^d^	0.78
G7 STZ‐aflatoxin	1201 ± 71^a^	30.03	412 ± 26.16	256 ± 13.33	110 ± 6.61	137 ± 7.12	111 ± 6.06	1026 ± 55.1^a^	25.65	175^a^	4.38
G8 STZ‐ aflatoxin ‐BM	698 ± 42^b^	17.45	196 ± 13.96	69 ± 4.01	125 ± 7.69	98 ± 5.89	119 ± 6.97	607 ± 41.11^b^	15.18	91^c^	2.28

Abbreviations: BM, barley microgreen; STZ, streptozotocin. All values are represented as mean ± S.D.

*Note*: Means with different letters are significantly different (*p* < 0.05).

### 3.6 Chromosomal aberrations

Cytogenetic data showed frequencies of structural chromosomal aberrations, numerical chromosomal aberrations, and mitotic activity induced by both xenobiotics (aflatoxin and STZ) and the modulatory role of BM treatment in the bone marrow cells of the male albino rats, which are presented in Table 8. The chromatid gaps, deletions, breaks, and centromeric were the main types of chromosomal aberrations. Aflatoxin and STZ resulted in a significant increase in the chromosomal aberration relative to that of the healthy control group (G1). The BM treatments for the intoxicated rats ameliorated the xenobiotics harmful effect and improved the disturbances in the present parameter but not completely. The frequencies of chromosomal aberration in intoxicated groups were significant likewise to a previous study (Salah, Abdou, & Abdel‐Rahim, 2010). The mutagenic effect of aflatoxin and STZ was reported, as it induces chromosomal aberrations due to a possible clastogenic effect (Lla et al., 2008). They decreased the mitotic frequencies inferring their cytotoxic effect. The treatments with BM showed a decrease in these frequencies of chromosomal aberrations in aflatoxin‐ and STZ‐intoxicated groups. The mitotic activity of bone marrow cells was examined in the 8 experimental groups. The mitotic frequencies were reduced significantly in intoxicated animals and were elevated by BM treatment but were still less than that of the healthy control rats in the intoxicated groups.

**TABLE 8:** Structural chromosomal aberration induced in male bone marrow cells of rats

Treatment	Types of chromosomal aberration				Total chromosomal aberration (T.aber)	T. aber excluding gaps	Mitotic activity
	Gap	Deletion	Break	Centromeric			MI/10,000 cells
G1 control	4.79 ± 0.22	0.81 ± 0.051	0.62 ± 0.041	0.60 ± 0.036	6.82^c^	2.03^c^	16.71 ± 1.00^a^
G2 BM	4.42 ± 0.24	0.86 ± 0.49	0.59 ± 0.312	0.62 ± 0.032	6.49^c^	2.07^c^	16.78 ± 0.89^a^
G3 aflatoxin	11.31 ± 0.66	3.21 ± 1.41	2.61 ± 0.161	2.62 ± 0.110	19.75^a^	8.44^a^	9.50 ± 0.87^c^
G4 aflatoxin BM	6.06 ± 0.39	2.60 ± 0.114	1.57 ± 0.099	2.39 ± 0.121	12.62^b^	6.56^b^	12.58 ± 0.69^b^
G5 STZ	11.62 ± 0.81	3.10 ± 0.172	2.58 ± 0.168	2.56 ± 0.162	19.86^a^	8.24^a^	9.54 ± 0.54^c^
G6 STZ‐BM	5.70 ± 0.32	2.07 ± 0.121	1.81 ± 0.111	2.14 ± 0.172	11.72^b^	6.02^b^	12.87 ± 0.78^b^
G7 STZ‐aflatoxin	12.00 ± 0.81	3.31 ± 0.201	3.19 ± 0.210	3.18 ± 0.182	21.68^a^	9.68^a^	9.44 ± 0.57^c^
G8 STZ‐aflatoxin‐BM	6.90 ± 0.39	2.85 ± 0.184	1.90 ± 0.111	1.80 ± 0.112	13.45^b^	6.55^b^	11.96 ± 0.81^bc^

Abbreviations: BM, barley microgreen; STZ, streptozotocin. All values are represented as mean ± S.D.

*Note*: Means with different letters are significantly different (*p* < 0.05).

### 3.7 Histopathological findings

**FIGURE 3:**
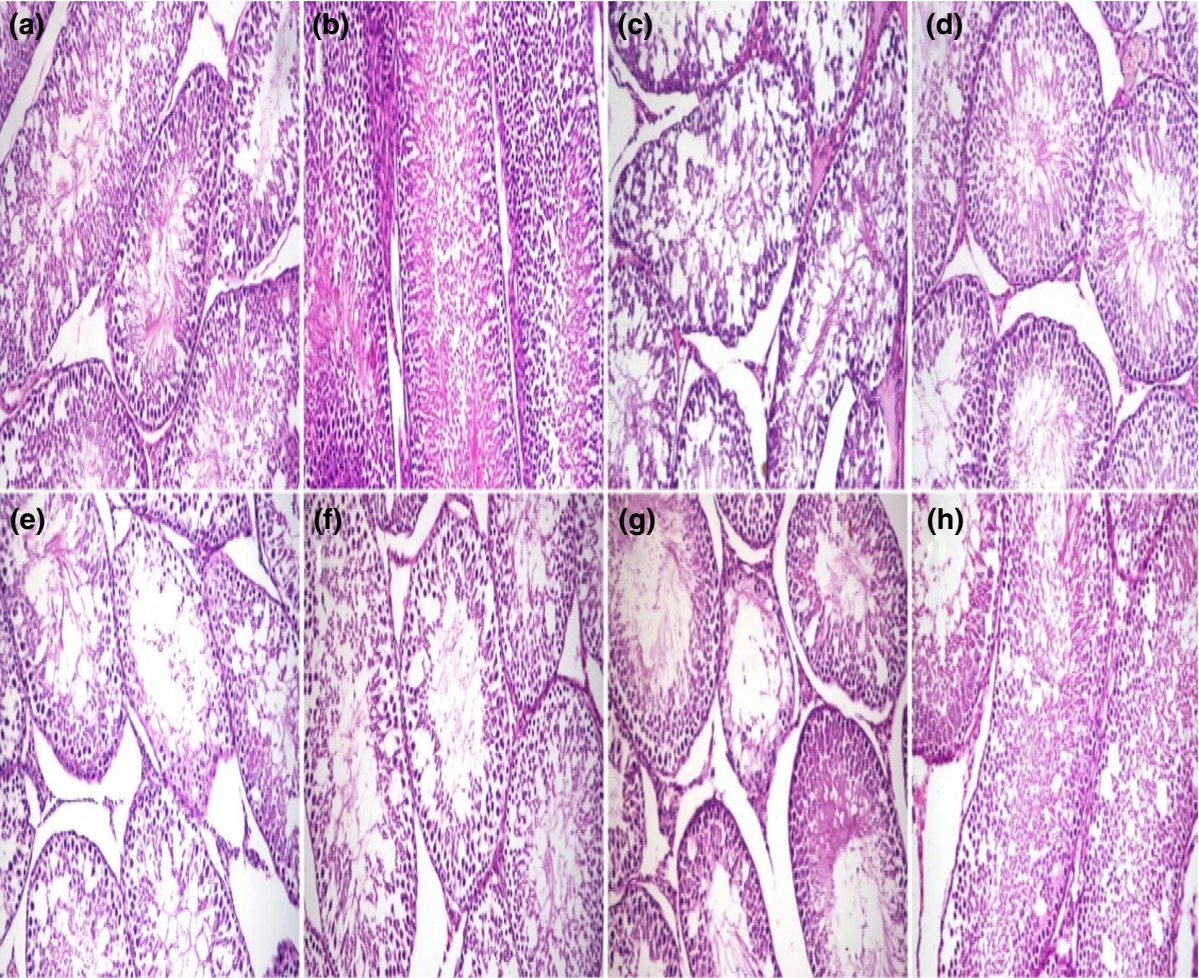
Histopathology of rat testis. (a) Well‐organized seminiferous tubules with normal germinal epithelium in G1 (control) and (b) G2 (BM group). (c) Moderate diffuse degeneration, decrease in the thickness of lining epithelium, and vacuolation of Sertoli cells in G3 (aflatoxin group). (d) Mild degeneration of seminiferous tubules in G4 (aflatoxin‐BM group). (e) Thinning of the lining epithelium and desquamated spermatocytes and early spermatids in the lumen in G5, (f) partially alleviation of testicular lesions in G6, (g) severe diffuse degeneration in the seminiferous tubules and intraluminal infiltration of homogenous hyalinized eosinophilic material in G7 (STZ‐aflatoxin), (h) mild degeneration of seminiferous tubules in G8 (STZ‐aflatoxin and BM groups). H and E stain ×200

**FIGURE 4:**
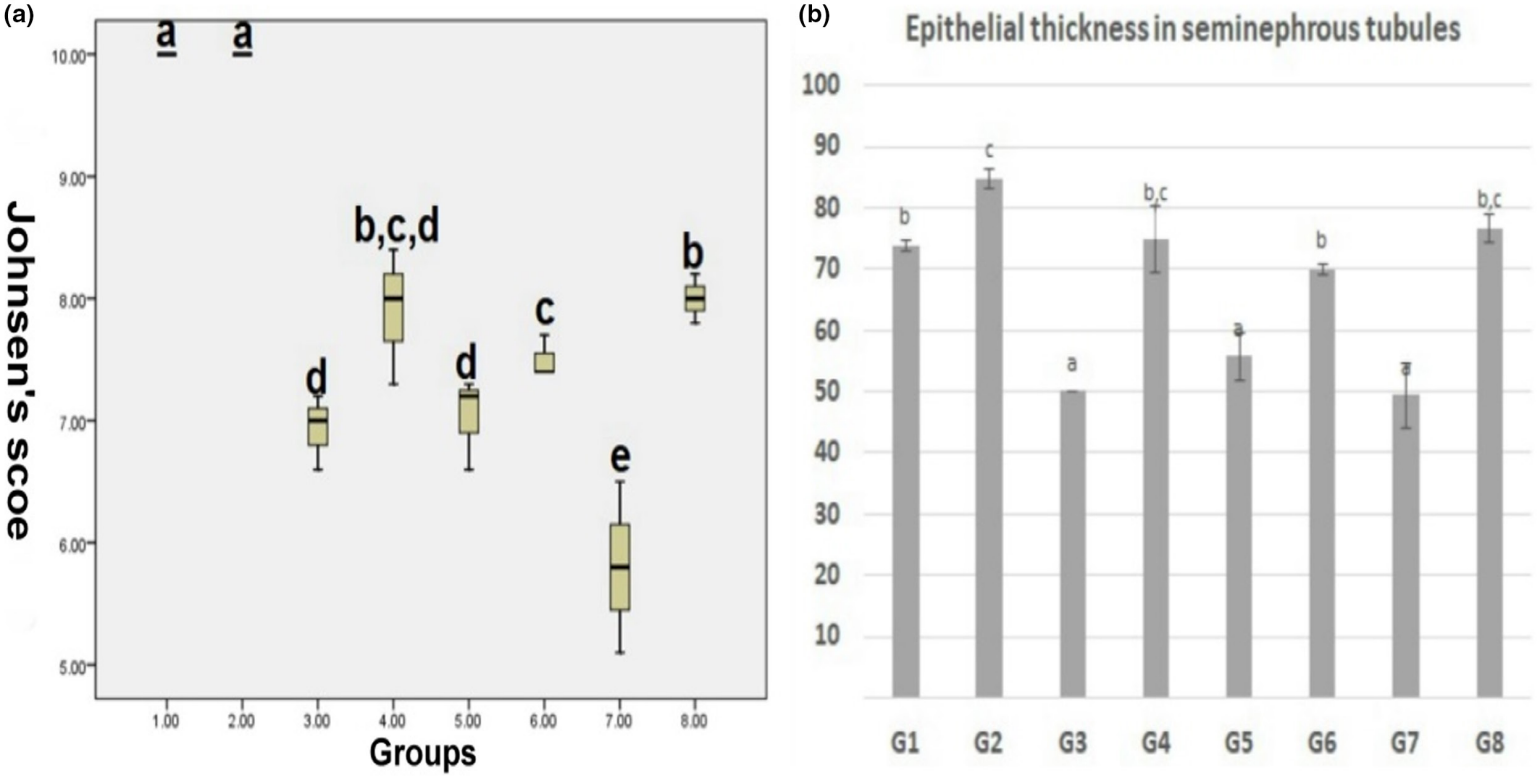
(a) Boxplot of Johnsen's score of spermatogenesis. The interquartile range (IQR) is represented by boxes. The medians are the thick middle lines. The thin horizontal lines at the top and bottom represent the maximum and minimum values. (b) Chart showing the epithelial thickness lining seminiferous tubules in different groups. Columns bearing different lowercase superscripts are significant at a *p*‐value ≤ 0.05

Microscopy of the testis in the control group and BM group revealed a normal histological structure in which multiple layers of spermatogenic cells lined the seminiferous tubules. In G3 (aflatoxin group), the seminiferous tubules showed moderate diffuse degeneration, a decrease in the thickness of lining epithelium, and vacuolation of Sertoli cells. In G4 (aflatoxin and BM group), the testis microscopy revealed mild degeneration of seminiferous tubules. In G5 (STZ group), some seminiferous tubules showed thinning of the lining epithelium and desquamated spermatocytes and early spermatids, and decreased spermatozoa in the lumen. In G6 (STZ‐BM group), the testicular lesions were partially alleviated compared with the previous group. In G7 (aflatoxin‐STZ group), the microscopy of the testes revealed severe diffuse degeneration in the seminiferous tubules and thinning of germinal epithelium. Intraluminal infiltration of homogenous hyalinized eosinophilic material was also noted in seminiferous tubules and moderate to a severe decrease in luminal spermatozoa. In G8 (STZ‐aflatoxin‐BM group), the degeneration of seminiferous tubules was reduced compared with G7 (Figure 3). According to Johnsen's score, spermatogenesis was impaired in G3, G5, and G7 and was partially restored in BM‐treated groups (G4, G6, and G8). However, it still recorded a significant decrease compared with the control group. The epithelial thickness lining seminiferous tubules was reduced in G3, G5, and G7, which was improved in BM‐treated groups (G4, G6, and G8). Similar studies also reported the reproductive disorders associated with aflatoxicosis and diabetes (La Vignera et al., 2012; Omur, Yildirim, Saglam, Comakli, & Ozkaraca, 2019). Increased oxidative stress in diabetes mellitus and aflatoxicosis was blamed for these disorders (WHO, 2018; Yigitturk et al., 2017; Supriya et al., 2014). Antioxidant diets like BM can attenuate the toxic effect of STZ and aflatoxin on sperm shape in rats (Salah et al., 2010; Lla et al., 2008; Omur et al., 2019; Narayna et al., 2005) likewise to the present finding (see Figure 4).

Barley microgreens alleviated the reproductive disorders, lipid profile, histopathology, and oxidative stress induced by aflatoxin and/or STZ. This beneficial effect of BM could be attributed to the high concentrations of vitamins, amino acids, enzymes, minerals, phenolics, antioxidants, and pigments: chlorophyll and carotenoids in the microgreen stage. Barley microgreens are a rich source of chlorophyll and carotenoids making them a potential candidate for pharmaceutical and nutraceutical use (Niroula et al., 2019).

In conclusion, aflatoxin and STZ exposure induced oxidative stress, decreased sperm count, increased sperm abnormalities, chromosomal aberration, and histopathological alteration in testis, which were improved by BM treatments. Subsequently, barley microgreen possesses an antioxidant activity making it a promising agent for protection against the xenobiotics' harmful oxidative stress.
